# Association between a metabolic score for insulin resistance and hypertension: results from National Health and Nutrition Examination Survey 2007–2016 analyses

**DOI:** 10.3389/fendo.2024.1369600

**Published:** 2024-04-22

**Authors:** Jing Zeng, Tingting Zhang, Yan Yang, Jinjing Wang, Dan Zheng, Yanwei Hou, Ye Tong, Xiaojing Fan, Xuan Wang, Yi Fang

**Affiliations:** Department of Endocrinology, Fifth Medical Center of Chinese PLA General Hospital, Beijing, China

**Keywords:** metabolic score for insulin resistance (METS-IR), insulin resistance, metabolic syndrome, hypertension, NHANES

## Abstract

**Background:**

The Metabolic Score for Insulin Resistance (METS-IR) offers a promising and reliable non-insulin-based approach to assess insulin resistance and evaluate cardiometabolic risk. However, evidence for the association between METS-IR and hypertension was still limited.

**Methods:**

Participants from the National Health and Nutrition Examination Survey (NHANES) database from 2007-2016 were selected for weighted multivariable regression analyses, subgroup analyses and restricted cubic spline (RCS) modeling to assess the association between the METS-IR and hypertension, as well as systolic blood pressure (SBP) and diastolic blood pressure (DBP).

**Results:**

This study enrolled 7,721 adults aged ≥20 years, 2,926 (34.03%) of whom was diagnosed as hypertension. After adjusting for all potential covariates, an increased METS-IR (log_2_ conversion, denoted as log_2_METS-IR) was independently associated with a higher prevalence of hypertension (odd ratio [OR] 3.99, 95% confidence interval [CI] 3.19~5.01). The OR for hypertension in subjects with the highest quartile of METS-IR was 3.89-fold (OR 3.89, 95% CI 3.06~4.94) higher than that in those with the lowest quartile of METS-IR. This positive correlation became more significant as METS-IR increased (*p* for trend < 0.001). Log_2_METS-IR was significantly correlated with increase in SBP (β 6.75, 95% CI 5.65~7.85) and DBP (β 5.59, 95% CI 4.75~6.43) in a fully adjusted model. Consistent results were obtained in subgroup analyses. Hypertension, SBP and DBP all exhibited a non-linear increase with the rise in METS-IR. The minimal threshold for the beneficial association of METS-IR with hypertension, SBP and DBP were all identified to be 46.88.

**Conclusion:**

The findings of this study revealed a significant positive association between METS-IR and hypertension among US adults, suggesting METS-IR as a potential tool for assessing hypertension risk.

## Introduction

Hypertension is a major risk factor for CVD, particularly ischemic heart disease and stroke. It has become a leading cause of global mortality and disability-adjusted life years (1–3). In 2010, about 31.1% of the global adult population (1.39 billion) had hypertension, comprising nearly 10% of worldwide healthcare spending (4). However, hypertension is not accompanied by obvious relevant symptoms, and patients can have hypertension without knowing it (5). Indeed, hypertension frequently coexists with a broader spectrum of anthropometric and metabolic abnormalities, encompassing abdominal (visceral) obesity, characteristic dyslipidemia (low high-density lipoprotein cholesterol and elevated triglyceride levels), glucose intolerance, insulin resistance, and hyperuricemia (6). Therefore, early identification and prevention of hypertension, along with comprehending its association with metabolic components is an essential issue.

Insulin resistance (IR) is closely linked to the substantial development and progression of diabetes (7–9). At present, the high insulin normoglycemic clamp (HEC) stands as the gold standard for assessing insulin sensitivity in peripheral tissues (10). Nonetheless, this invasive approach is intricate, time-consuming, and technically demanding, leading to the common preference for simpler indicators to assess insulin resistance. Traditional tools such as the homeostatic model assessment for IR (HOMA-IR) and quantitative insulin sensitivity check index (QUICKI), which use fasting insulin levels to measure insulin resistance, face practical limitations and variability (11). In addition to other insulin resistance assessment tools that do not require fasting insulin levels, including the product of glucose and triglycerides (TyG index), the product of glucose, triglycerides, and body mass index (TyG-BMI index), and the ratio of triglycerides divided by high density lipoprotein-cholesterol (TG/HDL-C ratio) (12–14), METS-IR emerges as an innovative tool for estimating insulin resistance. It employs readily available primary care parameters: fasting blood glucose (FBG), triglycerides (TG), high density lipoprotein-cholesterol (HDL-C), and body mass index (BMI). This approach eliminates the need for costly and variable fasting insulin tests. As a simple, indirect method, METS-IR identifies insulin resistance and corresponds with the underlying pathophysiological factors of metabolic syndrome, including obesity, dyslipidemia, hyperglycemia and intra-abdominal fat accumulation. Consequently, METS-IR also emerges as a promising metric for evaluating cardiometabolic risk (10, 15–17).

However, there are only few studies on the association between the METS-IR index and hypertension, with studies limited only to China, Mexico and Japan (15, 16, 18–22). The association between METS-IR and hypertension in the US population remains unclear. In this cross-sectional study, we aimed to explore the association between METS-IR and hypertension using data from the National Health and Nutrition Examination Survey (NHANES).

## Methods

### Study design and participants

Data were downloaded from the NHANES, a nationally representative cross-sectional survey designed and conducted by the National Center for Health Statistics (NCHS). The survey samples the US population using a stratified, multistage probability approach and offers health and nutrition statistics on the non-institutionalized civilian population in the United States. The NCHS Research Ethics Review Board authorized the survey, verifying that all participants provided informed consent. Detailed statistics are accessible at https://www.cdc.gov/nchs/nhanes/.

**Figure 1:**
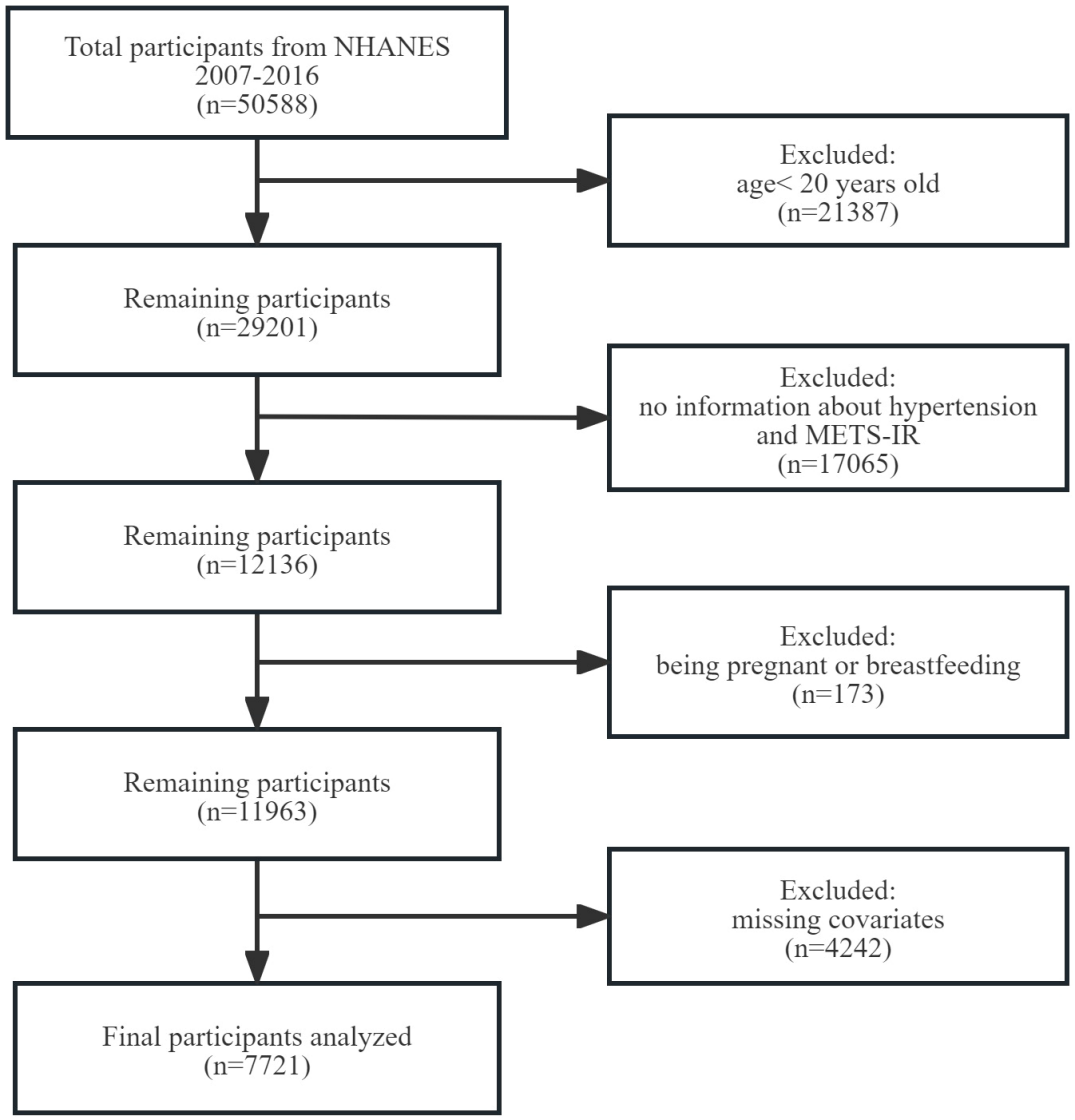
Flowchart of participant selection. NHANES, National Health and Nutrition Examination Survey. METS-IR, metabolic score for insulin resistance.

To evaluate the participants’ nutritional and physical health, standardized in-home interviews, physical examinations, and laboratory tests were carried out at mobile examination centers. 50,588 participants were involved in five NHANES cycles from 2007-2016. We excluded 21,387 participants under the age of 20 years, 17,065 with missing complete data about METS-IR and hypertension, 173 with pregnancy and breastfeeding, 4,242 with missing data of covariates. Eventually, 7,721 representative participants were enrolled in the study (**Figure 1**).

### Exposure variable

The arithmetic formula of METS-IR was (Ln[(2×FBG(mg/dL))+TG(mg/dL)]×BMI(kg/m²))/[Ln(HDL-C(mg/dL))] (23). After an 8.5-hour overnight fast, morning blood samples were collected to measure fasting glucose and total triglyceride levels. Enzymatic assays were utilized, and automated biochemical analyzers were employed to determine both triglyceride and fasting blood glucose concentrations. Serum triglyceride levels were assessed using Roche Modular P and Roche Cobas 6000 chemistry analyzers. Body mass index (BMI) was calculated as weight (kg) divided by height (m^2^).

### Outcome variable

Hypertension was defined using these criteria: (1) average SBP ≥140 mmHg, (2) average DBP ≥90 mmHg, (3) self-reported hypertension, or (4) the use of prescribed antihypertensive medications. These criteria adhere to the guidelines established by the International Society of Hypertension, with a threshold of 140/90 mmHg (24).

### Covariates

This study incorporated several covariates potentially affecting the association between METS-IR and hypertension. Demographic variables encompassed gender (male/female), age (years), race, educational attainment, family income, smoking and drinking habits, and physical activity levels. Biochemical parameters included uric acid (UA), total cholesterol (TC), and low-density lipoprotein cholesterol (LDL-C). Health risk factors comprised diabetes and cardiovascular disease (CVD). Racial/ethnic backgrounds were categorized into four groups: non-Hispanic white, non-Hispanic black, Mexican American, and other races. Educational attainment was stratified into three levels: less than 9 years, 9 to 12 years, and over 12 years of education. Family income was classified based on the poverty income ratio (PIR), as defined in a US government report. The categories for family income were defined as follows: low (PIR≤ 1.3), medium (PIR > 1.3 to ≤ 3.5), and high (PIR > 3.5). Smoking status was determined according to the criteria used in prior research, requiring a history of having smoked at least 100 cigarettes during one’s lifetime. Drinking status was assessed based on the consumption of a minimum of 12 alcoholic beverages within a year (25). Physical activity was quantified using Physical Activity Level (PAL) scores, which assess the intensity and frequency of various activities, including vigorous (2 points) or moderate (1 point) work-related activity, vigorous (2 points) or moderate (1 point) leisure-time physical activity, and walking or bicycling for transportation (1 point). PAL scores ranged from 0 (minimum) to 5 (maximum) (26). Based on these scores, we categorized physical activity as mild (PAL score 0-1), moderate (PAL score 2-3), or vigorous (PAL score 4-5). In our study, individuals were classified as diabetic based on any of the following: a self-reported diagnosis of diabetes from a doctor or health professional; self-reported use of insulin or diabetic pills; a fasting glucose concentration of ≥7.0 mmol/L; a 2-hour oral glucose tolerance test result of ≥11.1 mmol/L; or a glycohemoglobin HbA1c level of ≥6.5% (27). Cardiovascular disease (CVD) cases were identified through self-reported physician diagnoses, including congestive heart failure, coronary heart disease, angina, heart attack, or stroke (28, 29).

### Statistical analysis

For our statistical analyses, we followed the NHANES analytical guidelines, which are detailed at https://wwwn.cdc.gov/nchs/nhanes/tutorials/default.aspx. Our approach accounted for the survey’s complex sampling design, incorporating the Fasting Subsample 2 Year Mec Weight (WTSAF2YR×1/5). Baseline characteristics were stratified by quartiles of METS-IR. Continuous variables were presented as means ± standard error (SE), while categorical variables were expressed as percentages. To evaluate differences across the quartiles of METS-IR, we employed either the chi-squared test with Rao & Scott’s second-order correction or the Wilcoxon rank-sum test, both adapted for the analysis of complex survey samples. METS-IR was log_2_-transformed prior to regression analysis due to its right-skewed distribution. Weighted logistic regression was employed to evaluate the associations between METS-IR and hypertension, estimating the OR and 95% CI for each one-unit increase in log_2_METS-IR as well as for each METS-IR quartile. Additionally, weighted linear regression was used to assess the relationships between log_2_METS-IR and both SBP and DBP, estimating the regression coefficient (β) and its corresponding 95% CI. Five adjustment models were applied in the present study, with adjustment for potential confounders ascertained. Model 1 made no adjustments for covariates. Model 2 included adjustments for sex, age, race, education level, and family income. Model 3 incorporated additional adjustments for smoking status, drinking status, and physical activity. Model 4 extended the adjustments to include UA, TC, and LDL-C. Model 5 further expanded the adjustments to encompass diabetes and CVD.

We further explored potential modifications in the relationship between log_2_METS-IR and hypertension as well as SBP and DBP, considering variables such as sex, age (<65 vs. ≥65 years), race, education level (<9 years, 9~12 years, and ≥12 years), family income (low vs. medium or high), smoking status (No vs. Yes), drinking status (No vs. Yes), physical activity (mild vs. moderate or vigorous), diabetes (No vs. Yes), and CVD (No vs. Yes). We assessed subgroup heterogeneity using weighted multivariate logistic regression and examined interactions between subgroups and METS-IR through likelihood ratio testing.

Additionally, we employed RCS regression to evaluate non-linearity relationship between METS-IR and hypertension as well as SBP and DBP, following adjustment for variables in Model 5.

Since the sample size was determined based on available data, no prior statistical power calculation was conducted. We conducted our analyses using R software (version 4.3.3; R Foundation for Statistical Computing; http://www.Rproject.org), the R survey package (version 4.2.1), and Free Statistics software (version 1.9.2; Beijing Free Clinical Medical Technology Co., Ltd.). A two-sided p-value < 0.05 was considered statistically significant in all analyses. Data analysis was conducted from October 2023 to March 2024.

## Results

### Baseline characteristics of participants

In this study, out of a total of 50,588 patients, we included 7,721 adults aged 20 and above, representing a weighted population of 156,951,593 individuals. The cohort’s mean age, adjusted for the sample design, was 46.27 years (SE = 0.31), and it comprised 3,845 women, accounting for 49.65% of the weighted sample. Among these individuals, 2,926 (34.03%) were identified as having hypertension. The mean SBP and DBP were 120.37 (SE = 0.26) and 69.32 (SE = 0.26), respectively. METS-IR were significantly associated with all examined characteristics (all *p*<0.05). **Table 1** displays the baseline characteristics according to the METS-IR quartiles in a weighted analysis, whereas **Supplementary Table 1** presents the baseline characteristics by METS-IR quartiles in an unweighted analysis. To further validate the results, multiple imputations were conducted, with the distribution of baseline characteristics illustrated in **Supplementary Table 2**.

**Table 1:** Characteristics of the study population according to the quartiles of METS-IR, weighted^a^.

Characteristic	Overall N = 7721	Quartile 1 N = 1930	Quartile 2 N = 1930	Quartile 3 N = 1930	Quartile 4 N = 1931	*p* value
Sex, n (%)						<0.001
Male	3,876 (50.35)	753 (36.46)	1,009 (51.85)	1,095 (58.19)	1,019 (55.74)	
Female	3,845 (49.65)	1,177 (63.54)	921 (48.15)	835 (41.81)	912 (44.26)	
Age, Mean (SE)	46.27 (0.31)	44.19 (0.67)	47.68 (0.48)	47.46 (0.43)	45.87 (0.48)	<0.001
Race, n (%)						<0.001
Non-Hispanic White	3,649 (70.34)	955 (71.61)	926 (71.04)	856 (69.20)	912 (69.41)	
Non-Hispanic Black	1,354 (9.89)	319 (9.37)	343 (10.01)	343 (9.67)	349 (10.52)	
Mexican American	1,134 (7.82)	172 (4.83)	250 (6.70)	353 (9.79)	359 (10.17)	
Other races	1,584 (11.95)	484 (14.19)	411 (12.25)	378 (11.34)	311 (9.90)	
Education level (year), n (%)						<0.001
< 9	643 (4.50)	104 (2.63)	159 (4.65)	208 (5.80)	172 (5.04)	
9∼12	2,800 (32.40)	605 (27.75)	687 (31.57)	745 (35.63)	763 (34.97)	
≥12	4,278 (63.10)	1,221 (69.62)	1,084 (63.78)	977 (58.57)	996 (59.99)	
Family income, n (%)						<0.001
Low	2,404 (21.35)	557 (20.72)	556 (18.81)	599 (21.68)	692 (24.24)	
Medium	2,836 (35.22)	669 (31.33)	716 (35.69)	746 (37.35)	705 (36.74)	
High	2,481 (43.43)	704 (47.95)	658 (45.50)	585 (40.97)	534 (39.01)	
Smoking status, n (%)						0.027
No	4,256 (55.06)	1,140 (58.32)	1,053 (53.59)	1,042 (54.46)	1,021 (53.70)	
Yes	3,465 (44.94)	790 (41.68)	877 (46.41)	888 (45.54)	910 (46.30)	
Drinking status, n (%)						0.042
No	1,977 (20.96)	484 (19.73)	467 (19.30)	502 (21.57)	524 (23.31)	
Yes	5,744 (79.04)	1,446 (80.27)	1,463 (80.70)	1,428 (78.43)	1,407 (76.69)	
Physical activity, n (%)						<0.001
Mild	3,735 (43.65)	847 (38.04)	894 (41.02)	955 (45.87)	1,039 (50.00)	
Moderate	3,257 (45.78)	875 (50.22)	846 (47.36)	790 (43.20)	746 (42.07)	
Vigorous	729 (10.57)	208 (11.74)	190 (11.62)	185 (10.93)	146 (7.93)	
UA, umol/L, Mean (SE)	325.81 (1.27)	284.79 (2.38)	314.05 (2.30)	340.72 (1.89)	366.03 (2.25)	<0.001
TC, mmol/L, Mean (SE)	5.02 (0.02)	4.96 (0.03)	5.02 (0.03)	5.12 (0.03)	4.99 (0.03)	0.001
LDL-C, mmol/L, Mean (SE)	3.01 (0.01)	2.78 (0.03)	3.04 (0.02)	3.18 (0.03)	3.06 (0.03)	<0.001
Diabetes, n (%)						<0.001
No	6,957 (92.65)	1,851 (97.20)	1,791 (94.60)	1,717 (92.41)	1,598 (86.16)	
Yes	764 (7.35)	79 (2.80)	139 (5.40)	213 (7.59)	333 (13.84)	
CVD, n (%)						0.010
No	7,062 (93.02)	1,798 (94.19)	1,772 (93.28)	1,764 (93.72)	1,728 (90.87)	
Yes	659 (6.98)	132 (5.81)	158 (6.72)	166 (6.28)	203 (9.13)	
SBP, mmHg, Mean (SE)	120.37 (0.26)	116.01 (0.46)	120.18 (0.41)	121.70 (0.40)	123.81 (0.46)	<0.001
DBP, mmHg, Mean (SE)	69.32 (0.26)	66.83 (0.34)	68.29 (0.39)	70.29 (0.38)	72.01 (0.35)	<0.001
Hypertension, n (%)						<0.001
No	4,795 (65.97)	1,457 (79.97)	1,256 (68.03)	1,100 (61.49)	982 (53.61)	
Yes	2,926 (34.03)	473 (20.03)	674 (31.97)	830 (38.51)	949 (46.39)	
METS-IR, Mean (SE)	48.70 (0.26)	33.67 (0.10)	42.89 (0.06)	51.34 (0.07)	67.71 (0.35)	<0.001

^a^All means and SEs for continuous variables and percentages for categorical variables were weighted. SE, standard error; UA, uric acid; TC, total cholesterol; LDL-C, low density lipoprotein cholesterol; CVD, cardiovascular disease; SBP, systolic pressure; DBP, diastolic pressure; METS-IR, the Metabolic Score for Insulin Resistance.

### Associations of METS-IR with hypertension and SBP/DBP

**Table 2** displays the associations between the METS-IR and hypertension. Across all five adjusting models, METS-IR was positively associated with hypertension. The ORs for log_2_METS-IR, analyzed as a continuous variable, were consistently significant: OR 3.27 (95% CI 2.71~3.95, *p <*0.001) in Model 1; OR 4.46 (95% CI 3.65~5.45, *p* < 0.001) in Model 2; OR 4.46 (95% CI 3.64~5.47, *p* < 0.001) in Model 3; OR 4.08 (95% CI 3.26~5.10, *p* < 0.001) in Model 4; and OR 3.99 (95% CI 3.19~5.01, *p* < 0.001) in Model 5. We converted METS-IR from a continuous to a categorical variable (quartiles) for sensitivity analysis. In the fully adjusted Model 5, the adjusted ORs for hypertension in quartiles 2, 3, and 4 were 1.91 (95% CI 1.54~2.37), 2.7 (95% CI 2.15~3.40), and 3.89 (95% CI 3.06~4.94), respectively, using quartile 1 as the reference. This pattern of significant positive association persisted across all models (p < 0.001), underscoring a robust link between METS-IR levels and hypertension risk. Additionally, there was a significant increasing trend in hypertension risk across METS-IR quartiles (*p* for trend < 0.001, displayed in **Table 2**). Sensitivity analysis using multiple imputation of missing datasets corroborated these findings (see **Supplementary Table 3**).

**Table 2:** Association between METS-IR and hypertension.

	Model 1		Model 2		Model 3		Model 4		Model 5	
	OR (95% CI)	*p* value	OR (95% CI)	*p* value	OR (95% CI)	*p* value	OR (95% CI)	*p* value	OR (95% CI)	*p* value
Log_2_METS-IR	3.27 (2.71~3.95)	<0.001	4.46 (3.65~5.45)	<0.001	4.46 (3.64~5.47)	<0.001	4.08 (3.26~5.10)	<0.001	3.99 (3.19~5.01)	<0.001
METS-IR, Quartile										
Quartile 1	1(Ref)		1(Ref)		1(Ref)		1(Ref)		1(Ref)	
Quartile 2	1.88 (1.57~2.25)	<0.001	1.78 (1.45~2.19)	<0.001	1.78 (1.45~2.18)	<0.001	1.9 (1.53~2.37)	<0.001	1.91 (1.54~2.37)	<0.001
Quartile 3	2.5 (2.06~ 3.03	<0.001	2.64 (2.14~3.26)	<0.001	2.63 (2.13~3.24)	<0.001	2.68 (2.12~ 3.38)	<0.001	2.7 (2.15~3.40)	<0.001
Quartile 4	3.45 (2.84~4.21)	<0.001	4.43 (3.57~5.49)	<0.001	4.39 (3.53~ 5.45)	<0.001	3.97 (3.13~5.02)	<0.001	3.89 (3.06~4.94)	<0.001
Trend.test		<0.001		<0.001		<0.001		<0.001		<0.001

METS-IR, the Metabolic Score for Insulin Resistance; OR, odds ratio; CI, confidence interval; UA, uric acid; TC, total cholesterol; LDL-C, low density lipoprotein cholesterol; CVD, cardiovascular disease.

Model 1: No covariates were adjusted.

Model 2: Adjusted by sex, age, race, education level and family income.

Model 3: Adjusted by sex, age, race, education level, family income, smoking status, drinking status and physical activity.

Model 4: Adjusted by sex, age, race, education level, family income, smoking status, drinking status, physical activity, UA, TC and LDL-C.

Model 5: Adjusted by sex, age, race, education level, family income, smoking status, drinking status, physical activity, UA, TC, LDL-C, diabetes and CVD.

Across all regression models (**Table 3**), a significant positive association was observed between log_2_ METS-IR and both SBP and DBP. Specifically, in full adjusted model 5, the adjusted β for SBP was 6.75 (95% CI 5.65~7.85, P < 0.001) and the adjusted β for DBP was 5.59 (95% CI 4.75~6.43, p <0.001), indicating that each unit of increased log_2_ METS-IR was associated with 6.75 mmHg increased of SBP and 5.59 mmHg increased of DBP, respectively. This observation also persisted in other models (model 1, model 2, model 3 and model 4). Sensitivity analysis employing multiple imputation of missing datasets yielded consistent results (**Supplementary Table 4**), corroborating the observed associations.

**Table 3:** Association between METS-IR and SBP/ DBP.

	Model 1		Model 2		Model 3		Model 4		Model 5	
	β (95% CI)	*p* value	β (95% CI)	*p* value	β (95% CI)	*p* value	β (95% CI)	*p* value	β (95% CI)	*p* value
SBP	7.92 (6.80~ 9.03)	<0.001	6.48 (5.54~7.42)	<0.001	6.55 (5.57~7.53)	<0.001	6.98 (5.88~8.08)	<0.001	6.75 (5.65~7.85)	<0.001
DBP	5.14 (4.40~5.87)	<0.001	4.95 (4.26~5.63)	<0.001	4.97 (4.29~5.65)	<0.001	5.51 (4.68~6.34)	<0.001	5.59 (4.75~6.43)	<0.001

METS-IR, the Metabolic Score for Insulin Resistance; CI, confidence interval; SBP, systolic pressure; DBP, diastolic pressure; UA, uric acid; TC, total cholesterol; LDL-C, low density lipoprotein cholesterol; CVD, cardiovascular disease.

Model 1: No covariates were adjusted.

Model 2: Adjusted by sex, age, race, education level and family income.

Model 3: Adjusted by sex, age, race, education level, family income, smoking status, drinking status and physical activity.

Model 4: Adjusted by sex, age, race, education level, family income, smoking status, drinking status, physical activity, UA, TC and LDL-C.

Model 5: Adjusted by sex, age, race, education level, family income, smoking status, drinking status, physical activity, UA, TC, LDL-C, diabetes and CVD.

**Figure 2:**
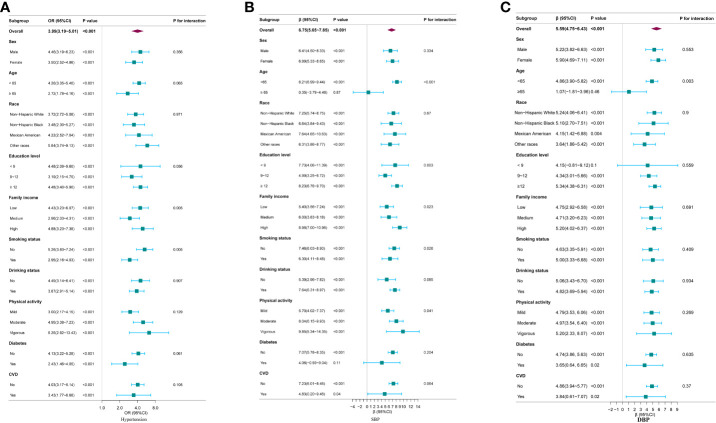
Subgroup analysis for the association between log_2_METS-IR and hypertension, as well as SBP and DBP. **(A)** Hypertension; **(B)** SBP; **(C)** DBP. Except for the stratification component itself,each stratification factor was adjusted for all other variables (sex, age, race, education level, family income, smoking status, drinking status, physical activity, UA, TC, LDL-C, diabetes and CVD).

Subgroup analyses revealed a consistent positive association between log_2_ METS-IR and hypertension across various subgroups. However, significant interaction effects were observed in family income and smoking status subgroups, indicating differential associations in these groups (*p* for interaction <0.05), as shown in **Figure 2A**. Regarding SBP, a significant positive association with log_2_METS-IR was detected in all subgroups with interaction effects observed in age, education level, family income, smoking status and physical activity subgroups (P for interaction <0.05), as depicted in **Figure 2B**. For DBP, a consistent positive association with log_2_METS-IR was observed across all groups, with interaction effects identified specifically within the age subgroups (P for interaction <0.05), illustrated in **Figure 2C**.

**Figure 3:**
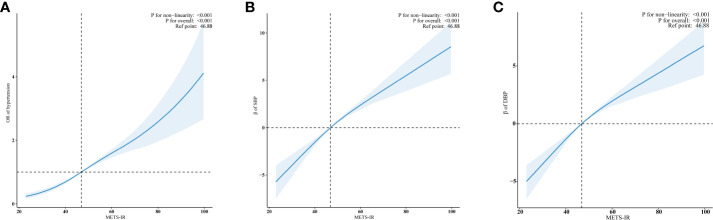
Examination of the dose-response relationship between log_2_METS-IR and hypertension, as well as SBP and DBP by RCS model. **(A)** Hypertension; **(B)** SBP; **(C)** DBP. The RCS model adjusted for sex, age, race, education level, family income, smoking status, drinking status, physical activity, UA, TC, LDL-C, diabetes and CVD. Only 99% of the data is shown.

Using RCS regression and adjusting for all covariates, we observed a significant positive non-linear relationship between METS-IR and hypertension risk, as well as SBP and DBP (all *p* for non-linearity < 0.001) (**Figures 3A-C**). The minimal thresholds for the beneficial association of METS-IR with hypertension (estimate OR =1), SBP (estimate β =0) and DBP (estimate β =0) were all 46.88.

## Discussion

In this population-based, cross-sectional study, we explored the association between METS-IR, an new tool for estimating insulin resistance, and hypertension. After adjusting for potential confounders, our results showed that METS-IR, either as a continuous or a categorical variable, was significantly associated with hypertension among US adults. This positive association also extended to the level of SBP and DBP. The subgroup analyses yielded similar results. Through RCS regression analysis, we observed a non-linear dose-response relationship between METS-IR and hypertension risk. The minimal thresholds for the beneficial association of METS-IR with hypertension, SBP and DBP were all 46.88. Our results indicate that METS-IR, readily assessable in primary care settings, could be an effective early marker for hypertension, contributing to primary prevention strategies.

Many studies have found that hypertension and insulin resistance were closely related and mutually causal. The well-established bidirectional link between hypertension and insulin resistance is substantiated by Wang et al.’s meta-analysis. This study found that elevated fasting insulin levels or insulin resistance, quantified using HOMA-IR, correlate with an increased risk of hypertension in the general population. Notably, individuals with the highest fasting insulin and HOMA-IR levels showed a 54% and 43% increased risk, respectively, of developing hypertension (30). Lin et al.’s study demonstrated that individuals with hypertension experienced a more significant increase in HOMA-IR over five years (ΔHOMA2-IR/5 yr) compared to non-hypertensive individuals (adjusted *p* = 0.044). Furthermore, those with treated hypertension were at the highest risk of developing diabetes, as evidenced by a hazard ratio (HR) of 2.98 (*p* < 0.001), and exhibited the greatest change in ΔHOMA2-IR/5 yr relative to those with normal blood pressure (31). Although some studies have identified a relationship between insulin resistance or hyperinsulinemia and hypertension, others, such as Ferrannini et al., have reported more ambiguous associations (32). Specifically, their study involving 2,241 normotensive, nondiabetic individuals showed that a substantial increase in plasma insulin concentration (200 μU/mL) was associated with only a minimal rise in blood pressure (BP) of 1 mm Hg. Moreover, other researchers have observed similar plasma insulin levels in normotensive, nondiabetic individuals and those with hypertension, hinting at a possible inverse relationship between insulin and BP (33). Furthermore, in various types of secondary hypertension not linked to obesity, such as renovascular or mineralocorticoid-induced hypertension, there is no evidence to suggest the presence of insulin resistance (34). These findings indicate that hyperinsulinemia or insulin resistance alone cannot fully account for a direct relationship with hypertension.

However, the metabolic outcomes of insulin resistance, including obesity, hyperglycemia, and dyslipidemia, may exacerbate hypertension (33). A novel measure for evaluating insulin resistance, named METS-IR, has been developed. This metric is calculated from fasting levels of glucose, triglycerides, HDL-C, and BMI. Crucially, METS-IR integrates aspects of obesity and metabolic syndrome and avoids dependence on fasting insulin levels (10). Several studies have investigated the relationship between METS-IR and hypertension. Han et al.’s research in normoglycemic individuals from Gifu, Japan, identified a significant link between high METS-IR and both pre-hypertension (adjusted OR = 1.95, 95% CI: 1.61–2.36) and hypertension (adjusted OR = 2.12, 95% CI: 1.44–3.11), persisting even after adjusting for confounders in multivariable logistic regression. Furthermore, when considering METS-IR as a continuous variable, each unit increase was associated with a 7% rise in pre-hypertension (adjusted OR = 1.07, 95% CI: 1.06–1.08) and a 13% increase in hypertension (adjusted OR = 1.13, 95% CI: 1.10–1.16). Stratified analyses showed a positive correlation between METS-IR and both pre-hypertension and hypertension across diverse normoglycemic subgroups (22). However, as the study data were sourced from Japanese subjects, the applicability of the findings to other ethnic groups remains uncertain. Additionally, this research, being a secondary analysis of pre-existing data, lacks clarity on the specific procedures used during medical consultations, such as the methodology for measuring blood pressure. Xu et al. explored the association between the METS-IR and hypertension in the non-overweight Chinese population. They observed a significant increase in the risk of developing hypertension in the third quartile group (HR 1.58, 95% CI 1.12–2.22), with an even higher risk in the fourth quartile group (HR 1.96, 95% CI 1.40–2.76), compared to the lowest quartile of METS-IR. The study identified a linear dose-response relationship between METS-IR and hypertension risk (HR 1.08, 95% CI 1.04–1.12) (35). The study failed to address key lifestyle influences like physical activity, alcohol consumption, and smoking, which are significant for blood pressure. Additionally, its findings, based primarily on Chinese participants, may not extend to other ethnic groups. Li et al. discovered that METS-IR served as a potent predictor of CVD and its subtypes among patients with hypertension and obstructive sleep apnea, thereby aiding in the identification of high-risk individuals and offering personalized CVD prevention strategies (15). In another study conducted by the same researchers, it was suggested that there exists an association between METS-IR and the risk of both overall stroke and ischemic stroke specifically among patients with hypertension (16). These two studies primarily concentrated on assessing the cardiovascular and cerebrovascular risks associated with METS-IR among patients with hypertension.

Our study, however, focused on exploring the association between METS-IR and hypertension among adults aged 20 years and older in the US, utilizing data from NHANES. We excluded BMI and waist circumference to avoid co-linearity with METS-IR, while included diabetes and CVD due to their known associations with hypertension. In our fully adjusted model, accounting for diabetes and CVD, we still found a robust association between METS-IR and hypertension risk, as well as SBP and DBP, even though prior research had already established the connections between METS-IR and both diabetes and CVD. Notably, our study reveals a dose-response relationship between METS-IR and hypertension, marking a significant finding in understanding hypertension’s metabolic drivers. We have established specific METS-IR thresholds at 46.88, linked to hypertension, SBP, and DBP, offering clinicians and researchers precise, actionable criteria for early detection and intervention. This development enhances hypertension risk stratification, incorporating METS-IR into assessments for a more refined prediction of hypertension risk. Such integration paves the way for personalized and more effective prevention strategies. Beyond its immediate findings, our study paves the way for future research into the mechanisms by which METS-IR influences hypertension. It opens up new avenues for exploring potential interventions that could mitigate this risk, thereby contributing to the broader goal of reducing the global burden of hypertension.

Pathophysiological evidence supports a link between METSIR and hypertension. Metabolic outcomes of insulin resistance may lead to hypertension through various mechanisms, including adipokines from fat tissue, altered gut microbiota, sympathetic nervous system (SNS) activation, imbalance in antinatriuretic and natriuretic hormones, and dysfunction in vascular and renal systems (36–39). Both animal and human studies suggest that hypertension in metabolic syndrome arises from factors that increase renal sodium reabsorption, leading to extracellular fluid volume expansion. Notably, three mechanisms are critical in this process: kidney compression by surrounding fat, renin-angiotensin-aldosterone system activation, and heightened SNS activity. Chronic obesity exacerbates hypertension and causes cardiovascular and renal damage, especially in conjunction with metabolic issues like hyperglycemia and hyperlipidemia (40, 41). Further investigation is needed to elucidate the exact underlying mechanisms and enhance our understanding of the pathophysiology of hypertension.

Several advantages can be attributed to our study. A representative sample of the US population is collected in the NHANES from 2007- 2016 based on a well-designed study protocol with extensive quality assurance and quality control. As a second step, we controlled for confounding covariates to ensure that our results are reliable and applicable to a broad range of individuals. We acknowledge, however, that the study has certain limitations. First, as a result of the cross-sectional nature of the study, we could not determine the temporal association between METS-IR and hypertension. Recognizing this limitation, we suggest that future research on the relationship between METS-IR and hypertension should include longitudinal designs. Such studies would enable researchers to track changes over time, providing clearer insights into whether elevated METS-IR levels precede the development of hypertension, thereby offering stronger evidence of a potential causal relationship. Second, because of a lack of data covariates, a large number of participants were excluded, which might cause bias. To mitigate this, we utilized multiple imputation (MI) techniques to address the gaps in our data, followed by a thorough re-analysis of the imputed dataset. Our sensitivity analysis shows that our primary conclusions remain stable, even when considering the potential impact of missing data, thus bolstering our confidence in the robustness of our findings. Third, the study did not eliminate bias from additional potential confounders, like dietary patterns, genetic predispositions, and psychosocial stressors, that were not adjusted for. Last, we recognize that relying on diagnoses derived from databases, instead of direct clinical measurements or diagnoses from medical institutions, introduces potential biases into our study. This limitation stems from the inherent nature of cross-sectional studies, which often depend on previously collected data and may lack the specificity and accuracy of clinical diagnoses. These limitations highlight the importance of conducting future longitudinal studies to investigate these aspects further.

## Conclusion

The findings of this cross-sectional study suggest that a higher METS-IR was independently associated with a higher prevalence of hypertension and a higher SBP and DBP. These findings indicate that METS-IR could potentially act as an effective tool for assessing hypertension risk and formulating targeted intervention strategies based on METS-IR levels. However, further longitudinal studies are necessary to validate these findings. Additional research is also needed to uncover the mechanisms through which METS-IR influences hypertension and to identify potential targets for therapy.

## Data availability statement

Publicly available datasets were analyzed in this study. This data can be found here: https://www.cdc.gov/nchs/nhanes/.

## Ethics statement

The studies involving humans were approved by The NCHS Research Ethics Review Board. The studies were conducted in accordance with the local legislation and institutional requirements. The participants provided their written informed consent to participate in this study. Written informed consent was obtained from the individual(s) for the publication of any potentially identifiable images or data included in this article.

## Author contributions

JZ: Writing – review & editing, Writing – original draft. TZ: Writing – review & editing, Writing – original draft. YY: Writing – review & editing, Writing – original draft. JW: Writing – review & editing, Writing – original draft. DZ: Writing – review & editing. YH: Writing – review & editing. YT: Writing – review & editing. XF: Writing – review & editing. XW: Writing – review & editing, Writing – original draft. YF: Writing – review & editing, Writing – original draft.
