# Supplementary material for: Association between a metabolic score for insulin resistance and hypertension: results from National Health and Nutrition Examination Survey 2007–2016 analyses
Source: Front Endocrinol (Lausanne). 2024 Apr 22;15:1369600. doi: 10.3389/fendo.2024.1369600 (PMC11070536; doi:10.3389/fendo.2024.1369600)
Supplement: Supplementary file 1 [file Table_1.docx]

Supplementary Material

Article Title

Jing Zeng*, Tingting Zhang*, Yan Yang*, Jinjing Wang*, Dan Zheng, Yanwei Hou, Ye Tong, Xiaojing Fan

*** Correspondence:** Yi Fang: [fangyi5zhongxin@163.com](mailto:fangyi5zhongxin@163.com)

*** Correspondence:** Xuan Wang: [endocrine@163.com](mailto:endocrine@163.com)

**Supplementary Table 1** Baseline characteristics according to the quartiles of METS-IR, unweighted

| Characteristic | Overall  (n = 7721) | Quartile 1  (n = 1930) | Quartile 2  (n = 1930) | Quartile 3  (n = 1930) | Quartile 4  (n = 1931) | *p*-Value |
| --- | --- | --- | --- | --- | --- | --- |
| Sex, n (%) |  |  |  |  |  | < 0.001 |
| Male | 3876 (50.2) | 753 (39) | 1009 (52.3) | 1095 (56.7) | 1019 (52.8) |  |
| Female | 3845 (49.8) | 1177 (61) | 921 (47.7) | 835 (43.3) | 912 (47.2) |  |
| Age, Mean ± SD | 48.3 ± 17.5 | 45.9 ± 18.8 | 49.7 ± 17.7 | 50.3 ± 17.1 | 47.3 ± 15.8 | < 0.001 |
| Race, n (%) |  |  |  |  |  | < 0.001 |
| Non-Hispanic White | 3649 (47.3) | 955 (49.5) | 926 (48) | 856 (44.4) | 912 (47.2) |  |
| Non-Hispanic Black | 1354 (17.5) | 319 (16.5) | 343 (17.8) | 343 (17.8) | 349 (18.1) |  |
| Mexican American | 1134 (14.7) | 172 (8.9) | 250 (13) | 353 (18.3) | 359 (18.6) |  |
| Other races | 1584 (20.5) | 484 (25.1) | 411 (21.3) | 378 (19.6) | 311 (16.1) |  |
| Education level (year), n (%) |  |  |  |  |  | < 0.001 |
| < 9 | 643 ( 8.3) | 104 (5.4) | 159 (8.2) | 208 (10.8) | 172 (8.9) |  |
| 9~12 | 2800 (36.3) | 605 (31.3) | 687 (35.6) | 745 (38.6) | 763 (39.5) |  |
| ≥12 | 4278 (55.4) | 1221 (63.3) | 1084 (56.2) | 977 (50.6) | 996 (51.6) |  |
| Family income, n (%) |  |  |  |  |  | < 0.001 |
| Low | 2404 (31.1) | 557 (28.9) | 556 (28.8) | 599 (31) | 692 (35.8) |  |
| Medium | 2836 (36.7) | 669 (34.7) | 716 (37.1) | 746 (38.7) | 705 (36.5) |  |
| High | 2481 (32.1) | 704 (36.5) | 658 (34.1) | 585 (30.3) | 534 (27.7) |  |
| Smoking status, n (%) |  |  |  |  |  | < 0.001 |
| No | 4256 (55.1) | 1140 (59.1) | 1053 (54.6) | 1042 (54) | 1021 (52.9) |  |
| Yes | 3465 (44.9) | 790 (40.9) | 877 (45.4) | 888 (46) | 910 (47.1) |  |
| Drinking status, n (%) |  |  |  |  |  | 0.184 |
| No | 1977 (25.6) | 484 (25.1) | 467 (24.2) | 502 (26) | 524 (27.1) |  |
| Yes | 5744 (74.4) | 1446 (74.9) | 1463 (75.8) | 1428 (74) | 1407 (72.9) |  |
| Physical activity, n (%) |  |  |  |  |  | < 0.001 |
| Mild | 3735 (48.4) | 847 (43.9) | 894 (46.3) | 955 (49.5) | 1039 (53.8) |  |
| Moderate | 3257 (42.2) | 875 (45.3) | 846 (43.8) | 790 (40.9) | 746 (38.6) |  |
| Vigorous | 729 ( 9.4) | 208 (10.8) | 190 (9.8) | 185 (9.6) | 146 (7.6) |  |
| UA, umol/L, Mean ± SD | 326.4 ± 82.3 | 287.1 ± 71.9 | 314.4 ± 75.7 | 340.3 ± 78.1 | 363.8 ± 82.7 | < 0.001 |
| TC, mmol/L, Mean ± SD | 5.0 ± 1.0 | 5.0 ± 1.0 | 5.0 ± 1.1 | 5.1 ± 1.0 | 5.0 ± 1.0 | 0.001 |
| LDL-C, mmol/L, Mean ± SD | 3.0 ± 0.9 | 2.8 ± 0.9 | 3.0 ± 0.9 | 3.1 ± 0.9 | 3.1 ± 0.9 | < 0.001 |
| Diabetes, n (%) |  |  |  |  |  | < 0.001 |
| No | 6957 (90.1) | 1851 (95.9) | 1791 (92.8) | 1717 (89) | 1598 (82.8) |  |
| Yes | 764 ( 9.9) | 79 (4.1) | 139 (7.2) | 213 (11) | 333 (17.2) |  |
| CVD, n (%) |  |  |  |  |  | < 0.001 |
| No | 7062 (91.5) | 1798 (93.2) | 1772 (91.8) | 1764 (91.4) | 1728 (89.5) |  |
| Yes | 659 ( 8.5) | 132 (6.8) | 158 (8.2) | 166 (8.6) | 203 (10.5) |  |
| SBP, mmHg, Mean ± SD | 122.1 ± 17.2 | 118.4 ± 17.9 | 122.1 ± 17.7 | 123.2 ± 16.7 | 124.6 ± 15.9 | < 0.001 |
| DBP, mmHg, Mean ± SD | 68.8 ± 12.5 | 66.6 ± 11.5 | 67.9 ± 12.5 | 69.3 ± 12.5 | 71.5 ± 12.9 | < 0.001 |
| Hypertension, n (%) |  |  |  |  |  | < 0.001 |
| No | 4795 (62.1) | 1457 (75.5) | 1256 (65.1) | 1100 (57) | 982 (50.9) |  |
| Yes | 2926 (37.9) | 473 (24.5) | 674 (34.9) | 830 (43) | 949 (49.1) |  |

SD, standard deviation; UA, uric acid; TC, total cholesterol; LDL-C, low density lipoprotein cholesterol; CVD, cardiovascular disease; SBP, systolic pressure; DBP, diastolic pressure; METS-IR, the Metabolic Score for Insulin Resistance

**Supplementary Table 2** Baseline characteristics according to the quartiles of METS-IR in weighted, imputed data via multiple imputation ^a^

| **Characteristic** | **Overall**  N = 11816 ^b^ | **Quartile 1**  N = 2954 | **Quartile 2**  N = 2954 | **Quartile 3**  N = 2954 | **Quartile 4**  N = 2954 | ***p* value** |
| --- | --- | --- | --- | --- | --- | --- |
| Sex, n (%) |  |  |  |  |  | <0.001 |
| Male | 5,849 (49.36%) | 1,174 (36.82%) | 1,558 (52.96%) | 1,623 (56.22%) | 1,494 (52.52%) |  |
| Female | 5,967 (50.64%) | 1,780 (63.18%) | 1,396 (47.04%) | 1,331 (43.78%) | 1,460 (47.48%) |  |
| Age, Mean (SE) | 47.56 (0.28) | 44.71 (0.62) | 48.66 (0.48) | 49.05 (0.38) | 48.07 (0.38) | <0.001 |
| Race, n (%) |  |  |  |  |  | <0.001 |
| Non-Hispanic White | 5,103 (67.35%) | 1,359 (68.97%) | 1,264 (67.73%) | 1,182 (65.55%) | 1,298 (67.00%) |  |
| Non-Hispanic Black | 2,272 (10.99%) | 509 (9.68%) | 577 (11.21%) | 580 (11.08%) | 606 (12.08%) |  |
| Mexican American | 1,825 (8.36%) | 273 (5.17%) | 431 (7.67%) | 571 (10.41%) | 550 (10.48%) |  |
| Other races | 2,616 (13.30%) | 813 (16.18%) | 682 (13.39%) | 621 (12.97%) | 500 (10.44%) |  |
| Education level (year), n (%) |  |  |  |  |  | <0.001 |
| < 9 | 1,338 (5.95%) | 216 (3.50%) | 359 (6.24%) | 412 (7.63%) | 351 (6.67%) |  |
| 9~12 | 4,392 (33.13%) | 985 (28.83%) | 1,068 (31.69%) | 1,142 (36.07%) | 1,197 (36.34%) |  |
| ≥12 | 6,086 (60.91%) | 1,753 (67.67%) | 1,527 (62.07%) | 1,400 (56.30%) | 1,406 (56.99%) |  |
| Family income, n (%) |  |  |  |  |  | <0.001 |
| Low | 3,954 (22.60%) | 928 (21.64%) | 907 (20.16%) | 983 (22.69%) | 1,136 (26.01%) |  |
| Medium | 4,418 (36.51%) | 1,048 (33.25%) | 1,114 (36.00%) | 1,146 (38.25%) | 1,110 (38.82%) |  |
| High | 3,444 (40.89%) | 978 (45.10%) | 933 (43.84%) | 825 (39.06%) | 708 (35.17%) |  |
| Smoking status, n (%) |  |  |  |  |  | 0.008 |
| No | 6,486 (55.19%) | 1,714 (57.89%) | 1,636 (55.40%) | 1,613 (54.65%) | 1,523 (52.59%) |  |
| Yes | 5,330 (44.81%) | 1,240 (42.11%) | 1,318 (44.60%) | 1,341 (45.35%) | 1,431 (47.41%) |  |
| Drinking status, n (%) |  |  |  |  |  | <0.001 |
| No | 3,347 (23.06%) | 793 (21.64%) | 795 (20.54%) | 876 (24.45%) | 883 (25.76%) |  |
| Yes | 8,469 (76.94%) | 2,161 (78.36%) | 2,159 (79.46%) | 2,078 (75.55%) | 2,071 (74.24%) |  |
| Physical activity, n (%) |  |  |  |  |  | <0.001 |
| Mild | 6,250 (47.28%) | 1,398 (41.01%) | 1,504 (44.30%) | 1,611 (48.95%) | 1,737 (55.37%) |  |
| Moderate | 4,608 (43.25%) | 1,255 (47.57%) | 1,184 (44.91%) | 1,118 (42.01%) | 1,051 (38.13%) |  |
| Vigorous | 958 (9.48%) | 301 (11.43%) | 266 (10.78%) | 225 (9.04%) | 166 (6.49%) |  |
| UA, umol/L, Mean (SE) | 327.18 (1.11) | 284.81 (1.89) | 317.92 (1.93) | 342.27 (1.96) | 367.30 (1.94) | <0.001 |
| TC, mmol/L, Mean (SE) | 4.99 (0.01) | 4.94 (0.03) | 4.99 (0.03) | 5.04 (0.03) | 4.98 (0.03) | 0.003 |
| LDL-C, mmol/L, Mean (SE) | 2.95 (0.01) | 2.76 (0.02) | 3.01 (0.02) | 3.10 (0.02) | 2.93 (0.02) | <0.001 |
| Diabetes, n (%) |  |  |  |  |  | <0.001 |
| No | 8,563 (78.76%) | 2,585 (90.82%) | 2,312 (84.20%) | 2,047 (76.66%) | 1,619 (62.36%) |  |
| Yes | 3,253 (21.24%) | 369 (9.18%) | 642 (15.80%) | 907 (23.34%) | 1,335 (37.64%) |  |
| CVD, n (%) |  |  |  |  |  | <0.001 |
| No | 10,489 (91.11%) | 2,745 (94.37%) | 2,648 (91.77%) | 2,617 (91.33%) | 2,479 (86.72%) |  |
| Yes | 1,327 (8.89%) | 209 (5.63%) | 306 (8.23%) | 337 (8.67%) | 475 (13.28%) |  |
| SBP, mmHg, Mean (SE) | 121.23 (0.25) | 116.77 (0.44) | 120.40 (0.42) | 122.79 (0.40) | 125.35 (0.36) | <0.001 |
| DBP, mmHg, Mean (SE) | 69.38 (0.24) | 67.03 (0.31) | 68.36 (0.35) | 70.37 (0.36) | 71.96 (0.30) | <0.001 |
| Hypertension, n (%) |  |  |  |  |  | <0.001 |
| No | 6,738 (62.32%) | 2,161 (78.13%) | 1,793 (66.32%) | 1,558 (58.73%) | 1,226 (44.82%) |  |
| Yes | 5,078 (37.68%) | 793 (21.87%) | 1,161 (33.68%) | 1,396 (41.27%) | 1,728 (55.18%) |  |
| METS-IR, Mean (SE) | 50.06 (0.27) | 34.02 (0.09) | 43.84 (0.06) | 52.88 (0.07) | 70.79 (0.37) | <0.001 |

^a^ All means and SEs for continuous variables and percentages for categorical variables were weighted. ^b^ 11,816 adults represent a weighted population of 208,245,759 individuals. SE, standard error; UA, uric acid; TC, total cholesterol; LDL-C, low density lipoprotein cholesterol; CVD, cardiovascular disease; SBP, systolic pressure; DBP, diastolic pressure; METS-IR, the Metabolic Score for Insulin Resistance

Supplementary Table 3 Association between METS-IR and hypertension by multiple imputation of missing datasets, weighted.

|  | Model 1 | | Model 2 | | Model 3 | | Model 4 | | Model 5 | |
| --- | --- | --- | --- | --- | --- | --- | --- | --- | --- | --- |
|  | OR (95% CI) | *p* value | OR (95% CI) | *p* value | OR (95% CI) | *p* value | OR (95% CI) | *p* value | OR (95% CI) | *p* value |
| log2METS-IR | 3.87 (3.35~4.47) | <0.001 | 4.88 ( 4.26~5.59) | <0.001 | 4.85 (4.22~5.58) | <0.001 | 4.06 (3.48~4.74) | <0.001 | 3.61 ( 3.06~4.25) | <0.001 |
| METS-IR  quartiles |  |  |  |  |  |  |  |  |  |  |
| Q1 | 1(Ref) |  | 1(Ref) |  | 1(Ref) |  | 1(Ref) |  | 1(Ref) |  |
| Q2 | 1.81 (1.55~ 2.12) | ＜0.001 | 1.65 (1.38~1.97) | ＜0.001 | 1.65 (1.38~1.97) | <0.001 | 1.67 (1.39~2.00) | ＜0.001 | 1.61 (1.35~1.92） | ＜0.001 |
| Q3 | 2.51 (2.16~ 2.92) | ＜0.001 | 2.45 (2.10~2.87) | ＜0.001 | 2.44 (2.10~2.84 | <0.001 | 2.32 (1.97~2.72) | ＜0.001 | 2.18 (1.86~2.56) | ＜0.001 |
| Q4 | 4.4 (3.76~ 5.14) | ＜0.001 | 5.3 (4.58~6.12) | ＜0.001 | 5.23 (4.50~6.08) | <0.001 | 4.33 (3.67~5.10) | ＜0.001 | 3.8 (3.21~4.50) | ＜0.001 |
| Trend test |  | <0.001 |  | <0.001 |  | <0.001 |  | <0.001 |  | <0.001 |

METS-IR, the Metabolic Score for Insulin Resistance; OR, odds ratio; CI, confidence interval; UA, uric acid; TC, total cholesterol; LDL-C, low density lipoprotein cholesterol; CVD, cardiovascular disease

Model 1: No covariates were adjusted

Model 2: Adjusted by sex, age, race, education level and family income

Model 3: Adjusted by sex, age, race, education level, family income, smoking status, drinking status and physical activity

Model 4: Adjusted by sex, age, race, education level, family income, smoking status, drinking status, physical activity, UA, TC and LDL-C

Model 5: Adjusted by sex, age, race, education level, family income, smoking status, drinking status, physical activity, UA, TC, LDL-C, Diabetes and CVD.

**Supplementary Table 4** Association between METS-IR and SBP/ DBP by multiple imputation of missing datasets, weighted.

|  | Model 1 | | Model 2 | | Model 3 | | Model 4 | | Model 5 | |
| --- | --- | --- | --- | --- | --- | --- | --- | --- | --- | --- |
|  | β (95% CI) | p value | β (95% CI) | p value | β (95% CI) | p value | β (95% CI) | p value | β (95% CI) | p value |
| SBP | 8.27 (7.26~9.28) | ＜0.001 | 6.38 (5.51~7.24) | ＜0.001 | 6.38 (5.51~7.26) | ＜0.001 | 5.75 (4.80~6.70) | ＜0.001 | 5.17 (4.17~6.17) | ＜0.001 |
| DBP | 4.82 (4.19~5.44) | ＜0.001 | 4.87 (4.27~5.48) | ＜0.001 | 4.87 (4.26~5.48) | ＜0.001 | 4.93 (4.28~5.59) | ＜0.001 | 4.92 (4.20~5.63) | ＜0.001 |

METS-IR, the Metabolic Score for Insulin Resistance; CI, confidence interval; SBP, systolic pressure; DBP, diastolic pressure; UA, uric acid; TC, total cholesterol; LDL-C, low density lipoprotein cholesterol; CVD, cardiovascular disease

Model 1: No covariates were adjusted

Model 2: Adjusted by sex, age, race, education level and family income

Model 3: Adjusted by sex, age, race, education level, family income, smoking status, drinking status and physical activity

Model 4: Adjusted by sex, age, race, education level, family income, smoking status, drinking status, physical activity, UA, TC and LDL-C

Model 5: Adjusted by sex, age, race, education level, family income, smoking status, drinking status, physical activity, UA, TC, LDL-C, Diabetes and CVD.
